# Gallbladder osseous metaplasia mimicking a focal lesion on ultrasound: a case report of a rare benign condition

**DOI:** 10.1093/jscr/rjag151

**Published:** 2026-03-17

**Authors:** Hazem Alouani, Salsabil Nasri, Amine Ben Safta, Mohamed Mahdi Trabelsi, Hichem Jerraya, Ramzi Nouira

**Affiliations:** Department of General Surgery B, Charles Nicolle Hospital – Boulevard du 9 avril 1938 Bab Saâdoun 1007 Tunis, Tunisia; Department of General Surgery B, Charles Nicolle Hospital – Boulevard du 9 avril 1938 Bab Saâdoun 1007 Tunis, Tunisia; Department of General Surgery B, Charles Nicolle Hospital – Tunis, Tunisia, Faculté de Médecine de Tunis, Université Tunis el Manar, Boulevard Avril 1938, Tunis, 1006, Tunis, Tunisia; Department of General Surgery B, Charles Nicolle Hospital – Boulevard du 9 avril 1938 Bab Saâdoun 1007 Tunis, Tunisia; Department of General Surgery B, Charles Nicolle Hospital – Tunis, Tunisia, Faculté de Médecine de Tunis, Université Tunis el Manar, Boulevard Avril 1938, Tunis, 1006, Tunis, Tunisia; Department of General Surgery B, Charles Nicolle Hospital – Tunis, Tunisia, Faculté de Médecine de Tunis, Université Tunis el Manar, Boulevard Avril 1938, Tunis, 1006, Tunis, Tunisia; Department of General Surgery B, Charles Nicolle Hospital – Boulevard du 9 avril 1938 Bab Saâdoun 1007 Tunis, Tunisia; Department of General Surgery B, Charles Nicolle Hospital – Tunis, Tunisia, Faculté de Médecine de Tunis, Université Tunis el Manar, Boulevard Avril 1938, Tunis, 1006, Tunis, Tunisia; Department of General Surgery B, Charles Nicolle Hospital – Boulevard du 9 avril 1938 Bab Saâdoun 1007 Tunis, Tunisia; Department of General Surgery B, Charles Nicolle Hospital – Tunis, Tunisia, Faculté de Médecine de Tunis, Université Tunis el Manar, Boulevard Avril 1938, Tunis, 1006, Tunis, Tunisia

**Keywords:** gallbladder, adenomyosis, osseous metaplasia, focal wall thickening, chronic cholecystitis

## Abstract

Focal gallbladder wall thickening may raise suspicion for inflammatory, hyperplastic, or malignant processes. We report a rare case of chronic cholecystitis associated with adenomyosis and osseous metaplasia in a 43-year-old woman with multiple comorbidities. The patient presented with right upper quadrant abdominal pain. Ultrasonography revealed focal gallbladder wall thickening without calculi. She underwent uneventful laparoscopic cholecystectomy. Gross and microscopic pathological examination demonstrated chronic cholecystitis with mucosal hyperplasia, intramural diverticula consistent with adenomyosis, and areas of osseous metaplasia, with no evidence of dysplasia or malignancy. The postoperative course was uncomplicated. Awareness of such rare benign lesions is essential, as they may clinically and radiologically mimic more aggressive disease.

## Introduction

Gallbladder wall thickening is a common radiological finding with a broad differential diagnosis, ranging from benign inflammatory lesions to gallbladder carcinoma [1]. Although chronic cholecystitis remains the most frequent cause, focal thickening often prompts further evaluation, as certain patterns may mimic malignancy. Adenomyosis (adenomyomatosis) is a benign hyperplastic condition characterized by mucosal invagination into the muscularis layer. Osseous metaplasia of the gallbladder is exceptionally rare, with only isolated descriptions in the literature [2].

In this report, we present a case of focal gallbladder wall thickening in a woman with a complex surgical history, where postoperative histology revealed chronic cholecystitis with adenomyosis and osseous metaplasia. This combination represents a rare benign process that may pose diagnostic challenges.

## Case report

**Figure 1:**
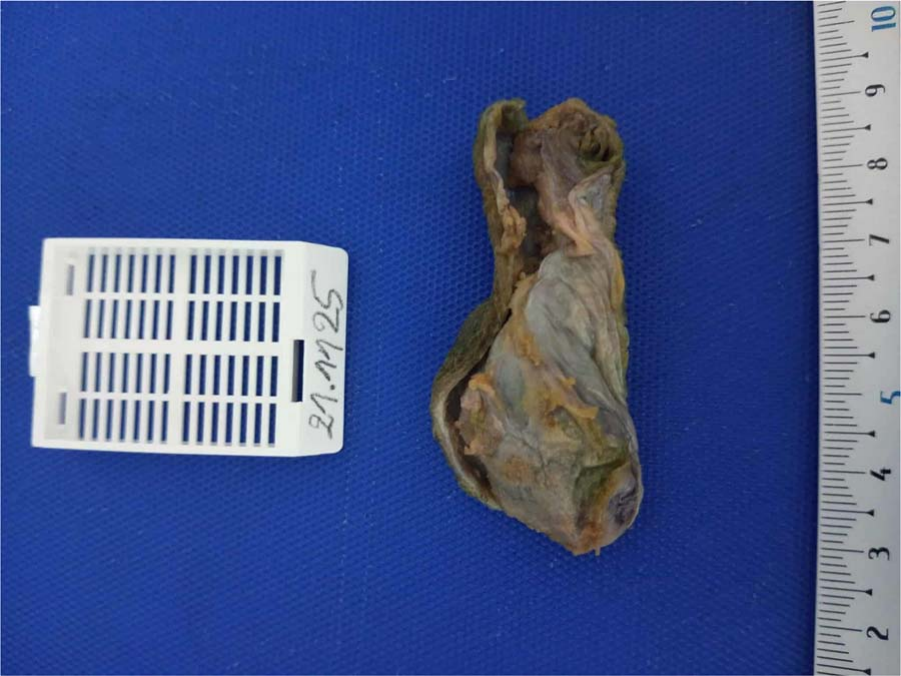
Macroscopic appearance of the gallbladder specimen gross photograph of the gallbladder measuring approximately 7 cm in length, opened longitudinally. The wall is thin with a focal area of mural thickening in the body region. The mucosa is smooth and greenish, without visible nodules, masses, or gallstones. This thickened area corresponded histologically to adenomyosis with osseous metaplasia.

A 43-year-old woman with type 2 diabetes and hypertension presented with intermittent right upper quadrant abdominal pain. Laboratory tests, including liver function parameters, were within normal limits. Abdominal ultrasonography demonstrated focal thickening of the gallbladder wall with preserved stratification, without gallstones or pericholecystic fluid (Fig. 1). Given the persistence of symptoms and localized thickening, laparoscopic cholecystectomy was indicated.

Intraoperatively, the gallbladder appeared slightly thickened over a focal area but showed no macroscopic signs of malignancy. The procedure was completed uneventfully, and the patient resumed oral intake on the first postoperative day. Gross examination revealed a 7.3 cm gallbladder, opened longitudinally, with overall thin walls and a focal thickened area in the body region. The mucosa was greenish and smooth, without nodules, masses, or gallstones. Multiple samples were taken from the thickened zone, cystic duct margin, and remaining segments.

**Figure 2:**
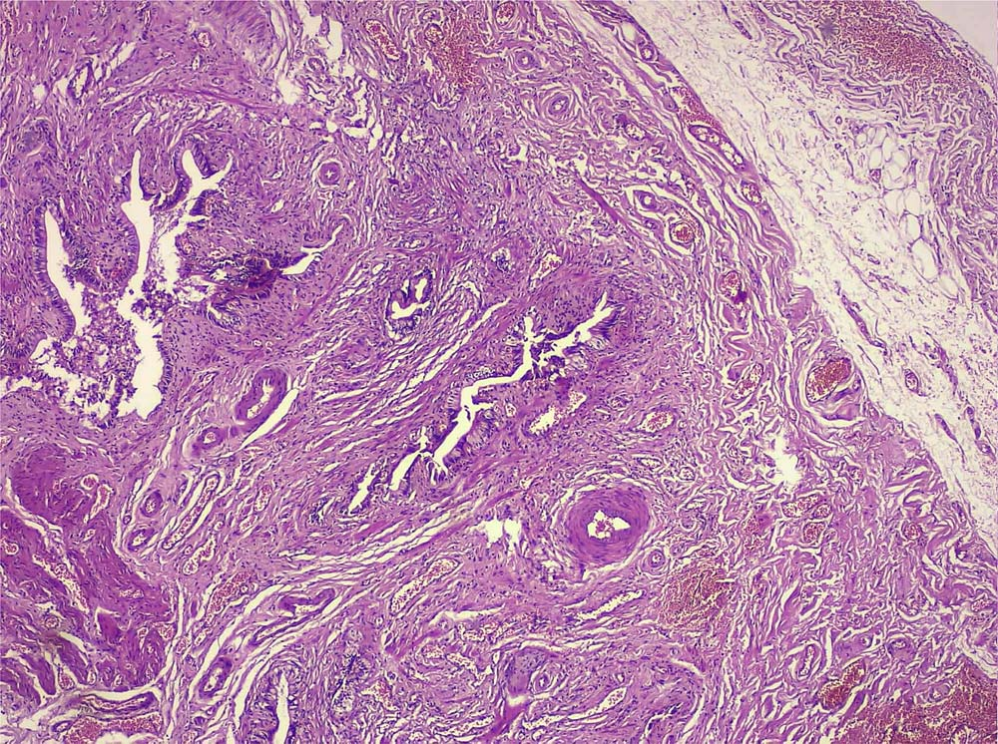
Low-magnification histology (H&E) low-power view demonstrating deep mucosal invaginations forming Rokitansky–Aschoff sinus–like diverticula extending into a hypertrophic muscular layer, consistent with adenomyosis. The surrounding stroma shows chronic inflammatory infiltrates without dysplasia or malignancy.

**Figure 3:**
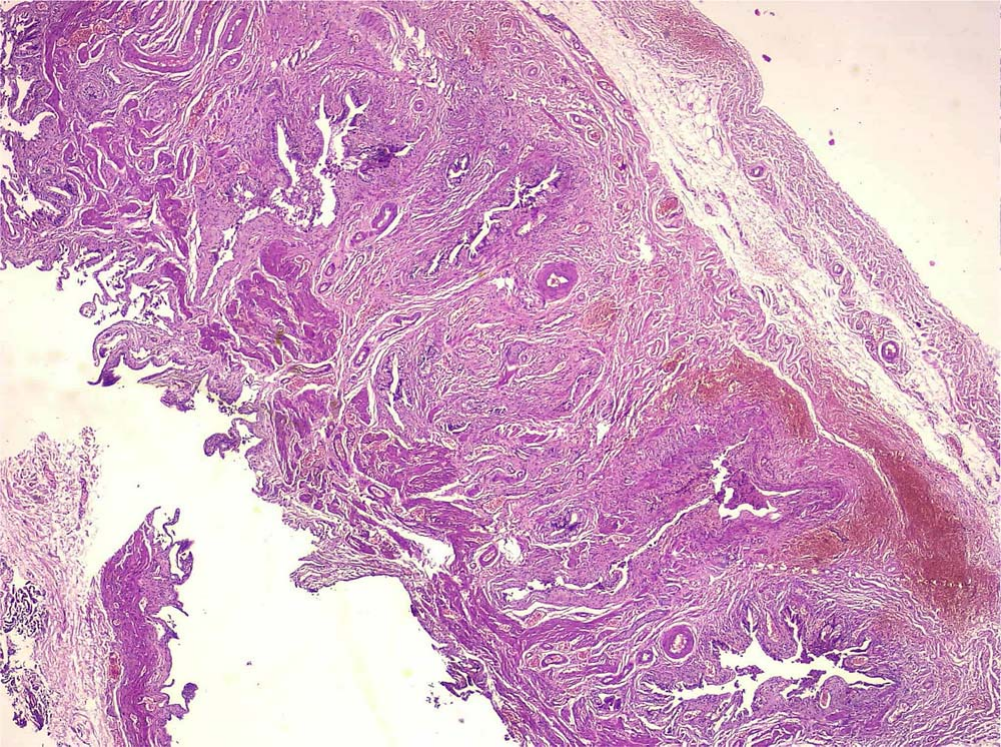
Medium-magnification histology (adenomyosis + osseous metaplasia zone, H&E) medium-power view showing complex architecture of deep mucosal glands and diverticula embedded within thickened smooth muscle bundles. Chronic lymphoplasmacytic inflammation is present, and trabecular bone consistent with osseous metaplasia is detected in deeper sections.

**Figure 4:**
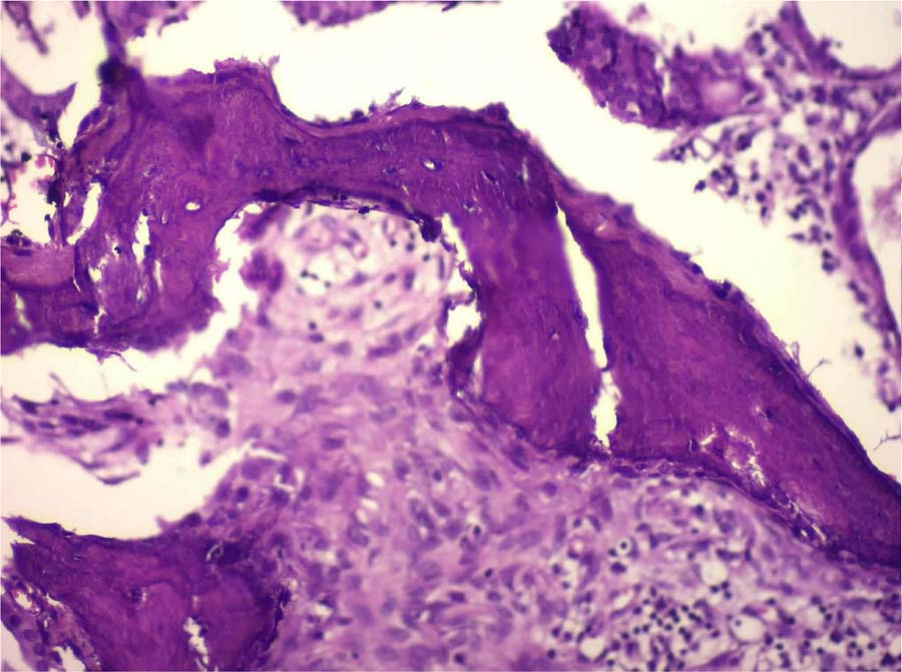
High-magnification histology showing osseous metaplasia (H&E) high-power photomicrograph demonstrating irregular trabeculae of eosinophilic osteoid matrix with embedded osteocyte-like cells, consistent with mature bone formation within adenomyosis. No atypia or malignancy is present.

Histological analysis showed mucosal invaginations forming Rokitansky–Aschoff sinus–like diverticula surrounded by hypertrophic smooth muscle bundles, consistent with adenomyosis. The stroma exhibited lymphoplasmacytic infiltrates, hypertrophied nerve fibers, and vascular dystrophy. In deeper sections, areas of vascularized granulation tissue contained trabecular bone formation, confirming osseous metaplasia. No dysplasia or neoplastic proliferation was identified (Figs 2–4).

The final diagnosis was chronic cholecystitis with adenomyosis and osseous metaplasia. The postoperative course was uneventful, and the patient was discharged without complications. Recognition of this rare benign entity is important to avoid misdiagnosis and unnecessary aggressive management.

## Discussion

Focal gallbladder wall thickening may represent a diagnostic dilemma, especially when imaging findings are nonspecific and calculi are absent. While chronic cholecystitis and adenomyomatosis are frequent benign causes, their radiological appearance may overlap with early gallbladder carcinoma, prompting surgical intervention [3, 4]. In particular, focal or asymmetric thickening, loss of mural stratification, or the presence of adjacent liver invasion on imaging raise concern for malignancy. However, even benign conditions such as localized adenomyomatosis, xanthogranulomatous cholecystitis, cholesterol polyps, and inflammatory pseudotumors may mimic these malignant features on ultrasound or CT. In our patient, the preservation of wall layering suggested a non-aggressive process, yet focal thickening without calculi kept the possibility of early carcinoma in the differential diagnosis.

Adenomyosis of the gallbladder is characterized by mucosal herniation into the muscular layer, forming Rokitansky–Aschoff sinuses [5]. Although generally benign, the focal type can cause localized thickening that mimics malignancy on ultrasound. Osseous metaplasia in the gallbladder is exceptionally rare, with fewer than a dozen cases reported in the literature, and likely reflects chronic inflammation driving stromal transformation and aberrant osteogenic activity [2]. Only isolated reports have described osseous metaplasia coexisting with chronic cholecystitis or adenomyomatosis, and none suggest a malignant potential [6]. The rarity of this phenomenon means radiological diagnosis is virtually impossible, making histopathology essential for accurate differentiation. Our case adds to the limited number of documented cases and reinforces that metaplastic bone formation can occur in the setting of chronic inflammation without neoplastic change.

Preoperative ultrasound in our patient demonstrated focal thickening without calculi, and malignancy could not be excluded. Cholecystectomy therefore remained the appropriate course of action. The presence of adenomyosis with osseous metaplasia underscores the heterogeneity of benign gallbladder pathology and the importance of thorough histopathological evaluation to exclude malignancy [7]. From a practical perspective, awareness of such rare benign entities is valuable for surgeons and radiologists, as it may help prevent unnecessary alarm, extensive workup, or overly aggressive operative strategies when imaging findings are ambiguous. Although surgery remains the standard of care in indeterminate cases, appreciating the spectrum of benign mimickers can guide appropriate counseling and postoperative reassurance. Our patient's postoperative course was uncomplicated, consistent with the benign nature of the condition.

## Conclusion

Chronic cholecystitis with adenomyosis and osseous metaplasia is a rare benign entity that may present as focal gallbladder wall thickening. Although imaging findings may mimic carcinoma, histopathological assessment remains definitive. Surgeons and radiologists should be aware of these unusual lesions to avoid diagnostic confusion and ensure appropriate management. Recognizing such rare benign mimickers also carries practical implications in reducing overtreatment and alleviating patient anxiety when indeterminate imaging features are encountered.
